# When a Giant Bulla Mimics a Tension Pneumothorax: A Case Report and Literature Review

**DOI:** 10.7759/cureus.88896

**Published:** 2025-07-28

**Authors:** Ramzi Addas, Wejdan Almrzouqi, Saud Alharbi, Shahad Almarwan

**Affiliations:** 1 Department of Thoracic Surgery, King Abdulaziz Medical City, National Guard Hospital, Jeddah, SAU; 2 Department of General Surgery, King Abdulaziz Medical City, National Guard Hospital, Jeddah, SAU; 3 College of Medicine, King Saud bin Abdulaziz University for Health Sciences, Jeddah, SAU; 4 College of Medicine, King Abdullah International Medical Research Center, Jeddah, SAU

**Keywords:** bullectomy, chest tube insertion, giant bullous emphysema, high-resolution computed tomography (hrct), tension pneumothorax, video-assisted thoracoscopic surgery (vats)

## Abstract

Giant bullous emphysema (GBE) is a rare condition marked by irreversible damage to the lung parenchyma. Its clinical presentation ranges from asymptomatic to various respiratory symptoms. GBE can closely resemble pneumothorax, and misdiagnosis may lead to inappropriate and potentially harmful interventions. A CT scan is essential to distinguish GBE from pneumothorax and to guide appropriate management. In this report, we present the case of a 38-year-old female who experienced shortness of breath for three months and was found to have a large left-sided bulla, confirmed by chest CT prior to any intervention. The patient underwent surgical management with favorable outcomes.

## Introduction

Giant bullous emphysema (GBE), also known as vanishing lung syndrome, is a rare yet significant form of chronic obstructive pulmonary disease (COPD), characterized by the presence of large bullae that occupy a substantial portion of the thoracic cavity and often compress adjacent lung parenchyma. First described by Burke in 1937, GBE typically involves giant air-filled spaces exceeding one-third of the hemithorax, most commonly affecting the upper lobes [1,2]. Pathophysiologically, these bullae result from progressive alveolar wall destruction, leading to localized areas of lung overdistention. These air spaces do not contribute to effective gas exchange and increase the work of breathing [3].

Although smoking is the most well-established risk factor for GBE, cases have also been reported in nonsmokers, suggesting additional genetic, environmental, or idiopathic factors may be involved [1]. COPD remains a major global public health concern, with substantial regional variation in prevalence, as evidenced by the comprehensive meta-analysis by Adeloye et al. [4] and region-specific studies such as those conducted in Saudi Arabia [5]. Despite the overall high prevalence of COPD, the true incidence of GBE is underreported due to its rarity and frequent misdiagnosis, particularly in acute care settings [6].

The clinical presentation of GBE is highly variable. Some patients remain asymptomatic for years, while others may present with progressive exertional dyspnea, hypoxia, recurrent respiratory infections, or chest discomfort [1]. In severe cases, massive bullae may exert a significant mass effect on mediastinal structures, leading to marked impairment of pulmonary function [7]. Additionally, the compressive effects of large bullae can cause atelectasis of the surrounding lung tissue, further contributing to respiratory insufficiency [8].

Radiological imaging plays a central role in the diagnosis of GBE. On chest radiographs, giant bullae (GBs) appear as large, lucent areas that can resemble pneumothorax due to the absence of vascular markings. However, high-resolution CT (HRCT) is the gold standard for definitive diagnosis, offering detailed visualization of the bullae, their relationship to surrounding lung parenchyma, and any concurrent pleural abnormalities [3]. The presence of thin-walled cystic spaces, often located in the upper lobes, is characteristic of GBE, and HRCT is crucial for distinguishing it from pneumothorax and other cystic lung diseases [7].

Management strategies for GBE depend on the severity of symptoms, size of the bullae, and degree of underlying lung function impairment. Asymptomatic or minimally symptomatic patients may be managed conservatively through regular monitoring and optimization of COPD treatment [9]. Conversely, patients with significant functional impairment or complications such as infection, hemorrhage, or pneumothorax may require surgical intervention. Bullectomy - particularly via minimally invasive techniques such as video-assisted thoracoscopic surgery (VATS) and, more recently, robotic-assisted surgery - has shown favorable outcomes in selected patients by re-expanding compressed lung tissue and improving respiratory mechanics [7,8]. Advances in uniportal VATS have further reduced surgical morbidity by allowing precise excision of bullae while preserving adjacent lung tissue [7].

One of the most critical clinical challenges in GBE is its frequent misdiagnosis as pneumothorax due to overlapping radiographic features. Such misinterpretation may lead to inappropriate chest tube placement, which can result in bulla perforation, prolonged air leaks, iatrogenic pneumothorax, and worsening respiratory status [6]. The “double-wall sign” on CT imaging - where both the inner and outer walls of a bulla are visible - is a key diagnostic feature that helps differentiate GBE from pneumothorax. In cases of diagnostic uncertainty, timely CT imaging is essential to guide appropriate management and avoid unnecessary invasive procedures [10].

Several reports have documented instances where large bullae were mistaken for pneumothorax, leading to inappropriate interventions. We present this case to underscore the importance of accurately distinguishing between GBE and pneumothorax in clinical practice.

## Case presentation

A 38-year-old female patient, medically and surgically free with no history of smoking, presented to our emergency department with a three-month history of shortness of breath, which had progressed to occur with mild exertion and eventually at rest. She reported no other symptoms or complaints. Prior to her presentation, she had been evaluated at another hospital, where she was diagnosed with a tension pneumothorax and was scheduled for chest tube insertion. However, the patient was hesitant and refused the procedure, opting to seek a second medical opinion.

**Figure 1:**
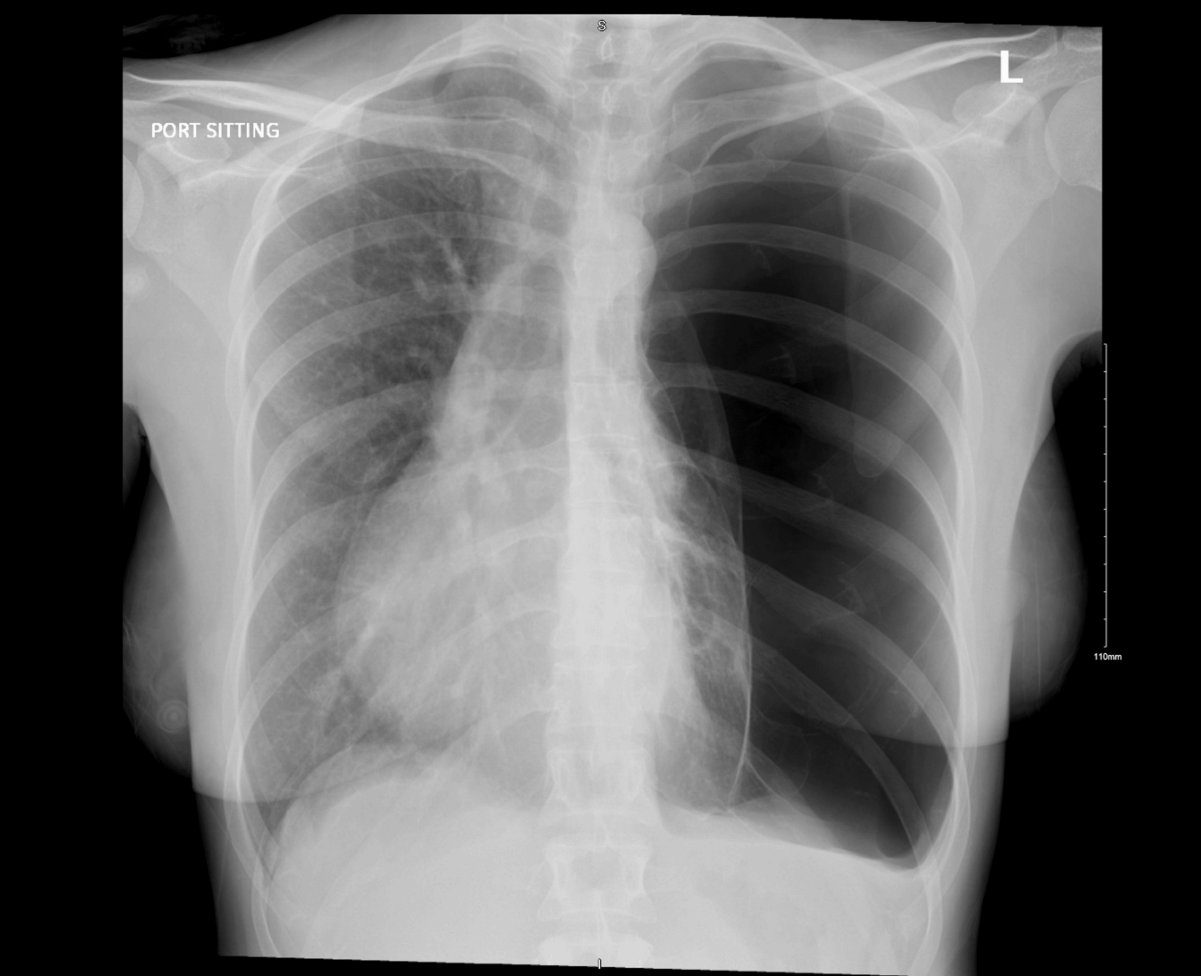
Preoperative chest X-ray showing a large bulla on the left side

**Figure 2:**
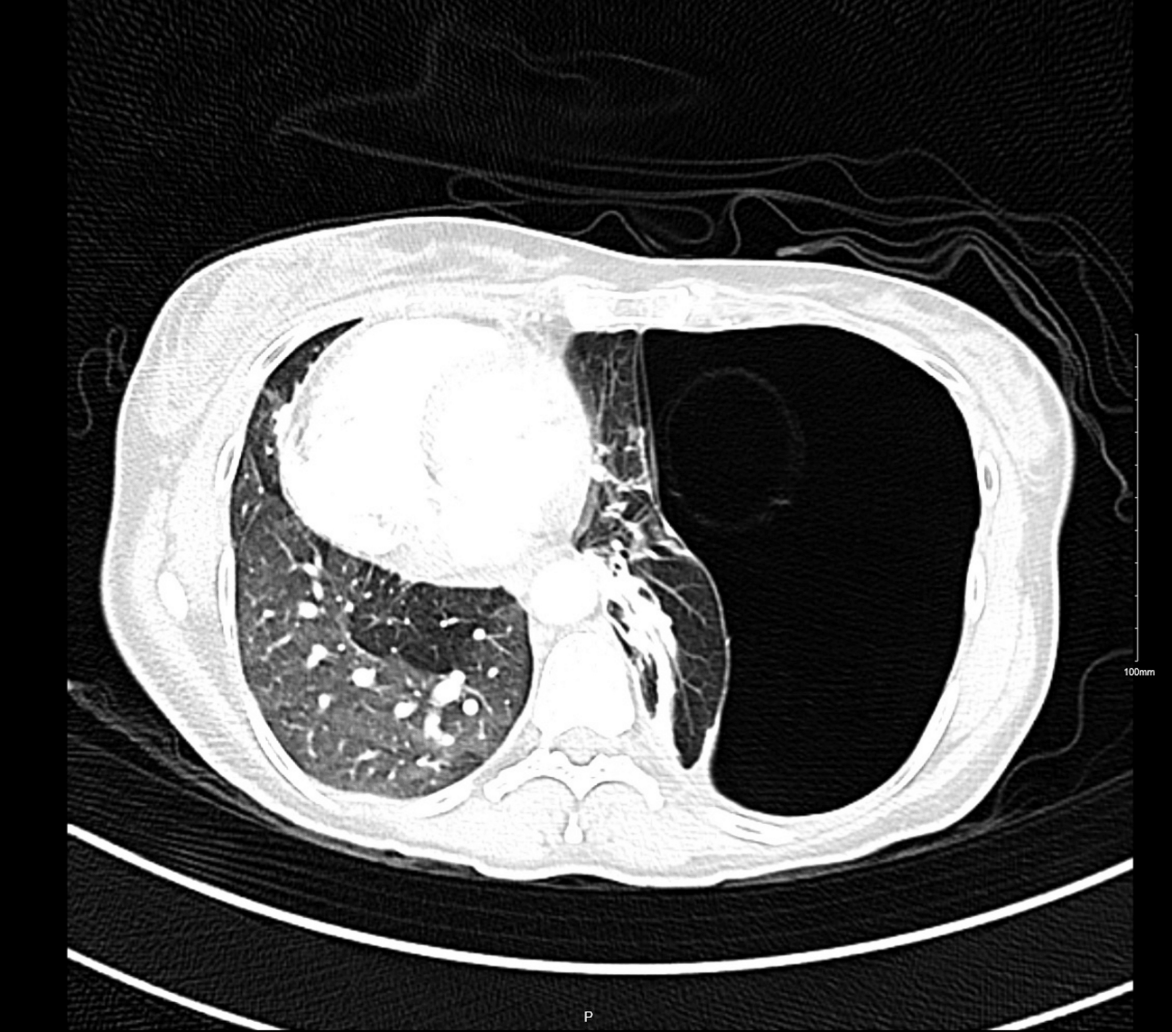
Chest CT scan revealing large bullae on the surface of the left lung, with marked mediastinal shift toward the contralateral side

On examination, she was vitally stable, with an oxygen saturation of 99% on room air. There was no chest wall deformity or tenderness, but auscultation revealed decreased air entry throughout the left lung field, with normal respiratory effort. A chest X-ray revealed a large left-sided bulla (Figure 1). Additional imaging was obtained to confirm the diagnosis before any intervention. A chest CT scan revealed a chronic, encysted, large bulla on the surface of the left lung, along with a marked mediastinal shift to the contralateral side (Figure 2).

The patient was admitted for close monitoring, underwent preoperative evaluation by the anesthesia team, and was scheduled for left VATS with bullectomy.

The patient agreed to undergo surgery after being informed of its benefits and potential complications. She was intubated with a left-sided double-lumen endotracheal tube and positioned in the right lateral decubitus position. A small transverse skin incision was made in the eighth intercostal space along the posterior axillary line. Dissection proceeded directly onto the rib with caution to avoid puncturing the bulla upon entry, allowing for proper assessment of its size and borders. A trocar was introduced through this incision, followed by the insertion of the thoracoscopic camera.

**Figure 3:**
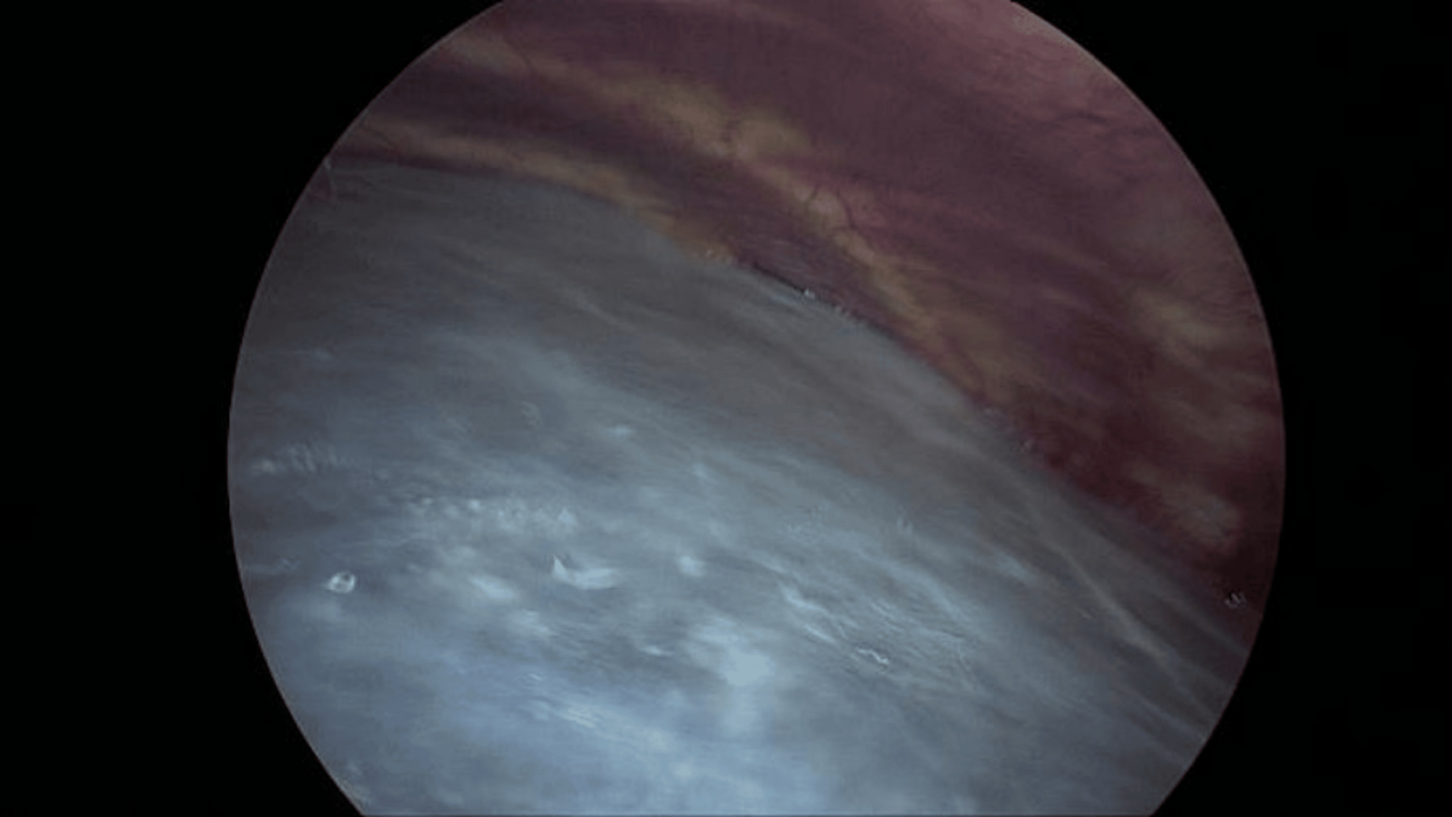
Intraoperative view showing a large bulla covering almost the entire surface of the left lung

Intraoperatively, a large bulla was visualized, covering almost the entire surface of the left lung (Figure 3). A second utility incision was made anteriorly at the level of the fourth intercostal space, and an Alexis retractor was applied. A third port was inserted posteriorly, approximately at the level of the fifth intercostal space.

**Figure 4:**
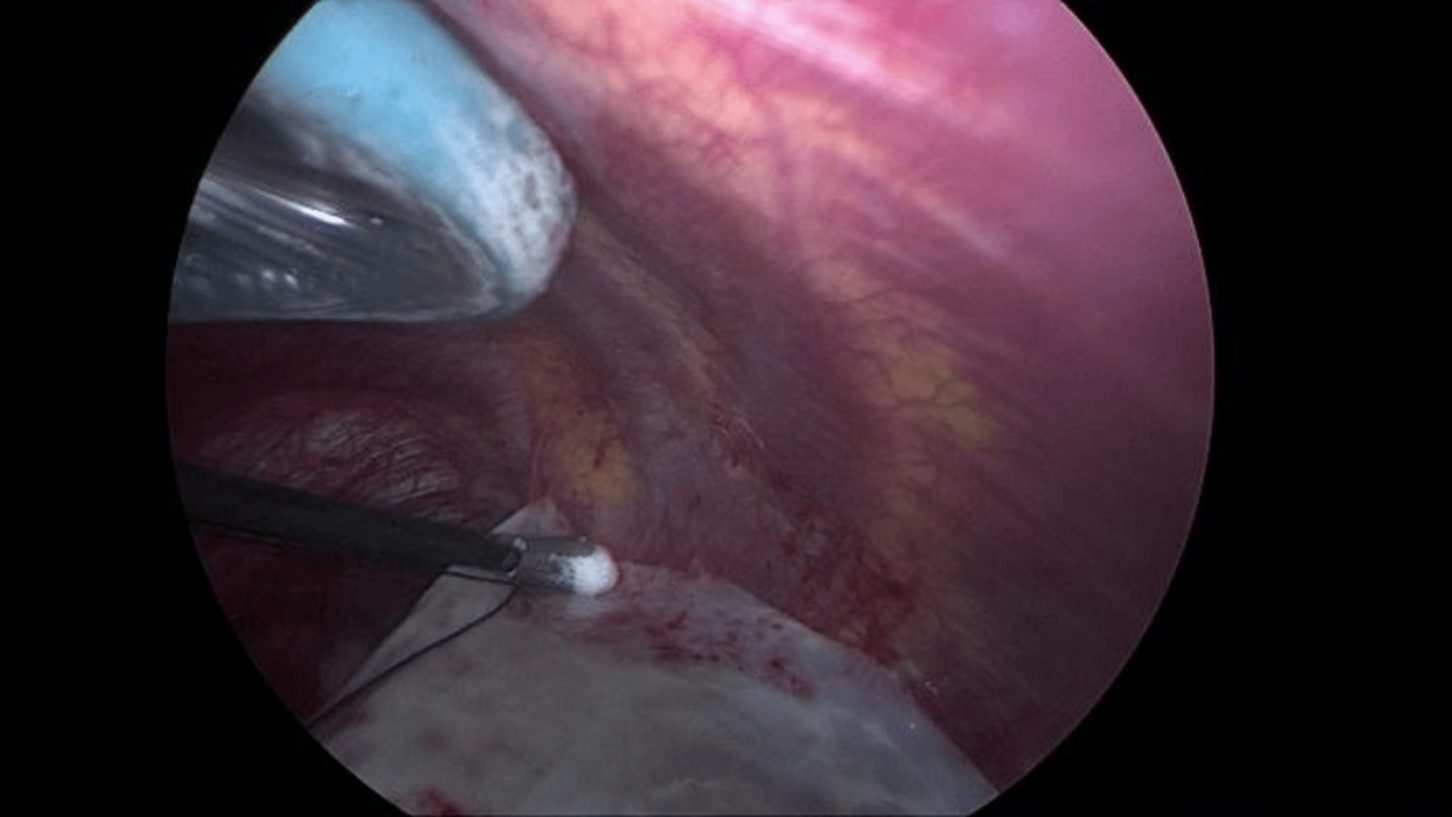
Blunt dissection used to free the bulla from flimsy adhesions to the chest wall

**Figure 5:**
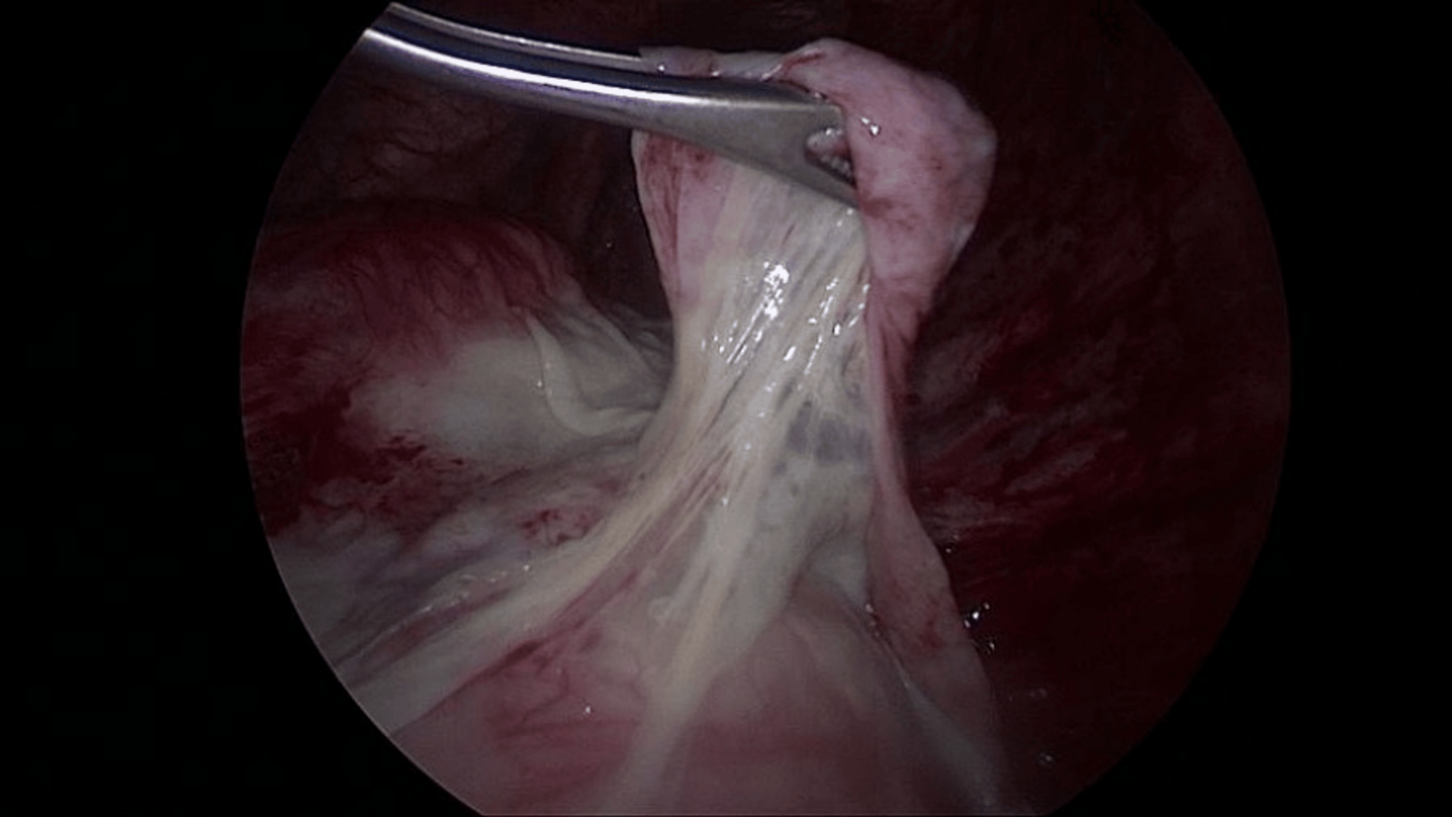
Intraoperative rupture of the bulla facilitated easier grasping and manipulation

Dissection of the bulla began, revealing flimsy adhesions to the chest wall that were easily separated with blunt dissection (Figure 4). During this process, the bulla ruptured, which facilitated easier manipulation (Figure 5). The borders of the lung became visible following the rupture. The entire bulla was dissected free without injury to the underlying lung. The area of attachment to the lung was clearly identified and resected using a 45-mm purple cartridge Endo-GIA stapler. The resected bulla was completely removed and sent for pathological examination. 

**Figure 6:**
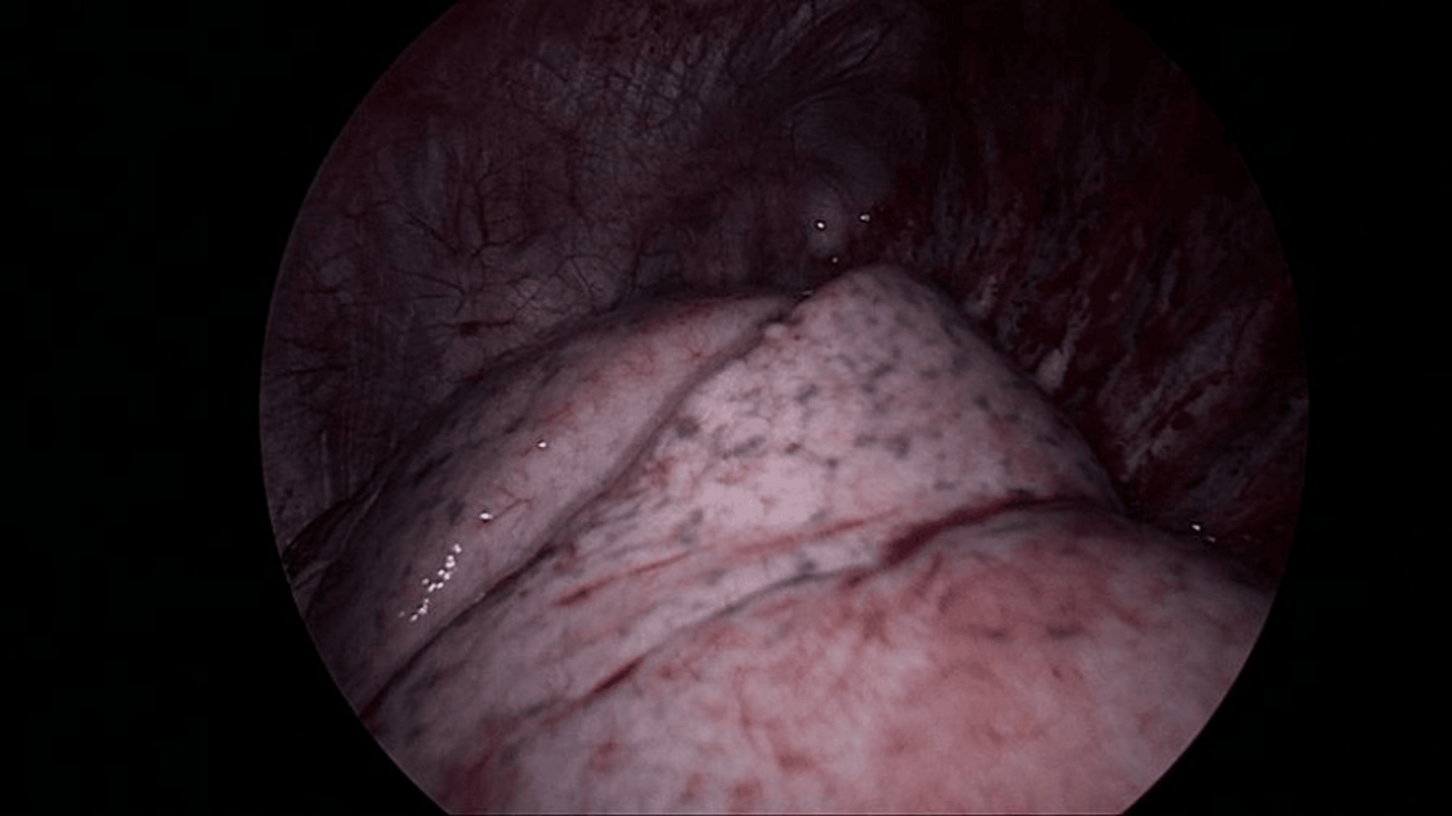
Intraoperative observation showing full inflation of the left lung with no air leak following bullectomy

The cavity was then lavaged with warm saline. The anesthesiologist resumed two-lung ventilation, and the left lung was observed to inflate fully without any air leak (Figure 6), which was reassuring in the context of bullous resection. A 28-French straight chest tube was placed through the posterior incision and secured in position. Hemostasis was confirmed to be satisfactory. The utility incision and camera port were closed in the usual fashion.

**Figure 7:**
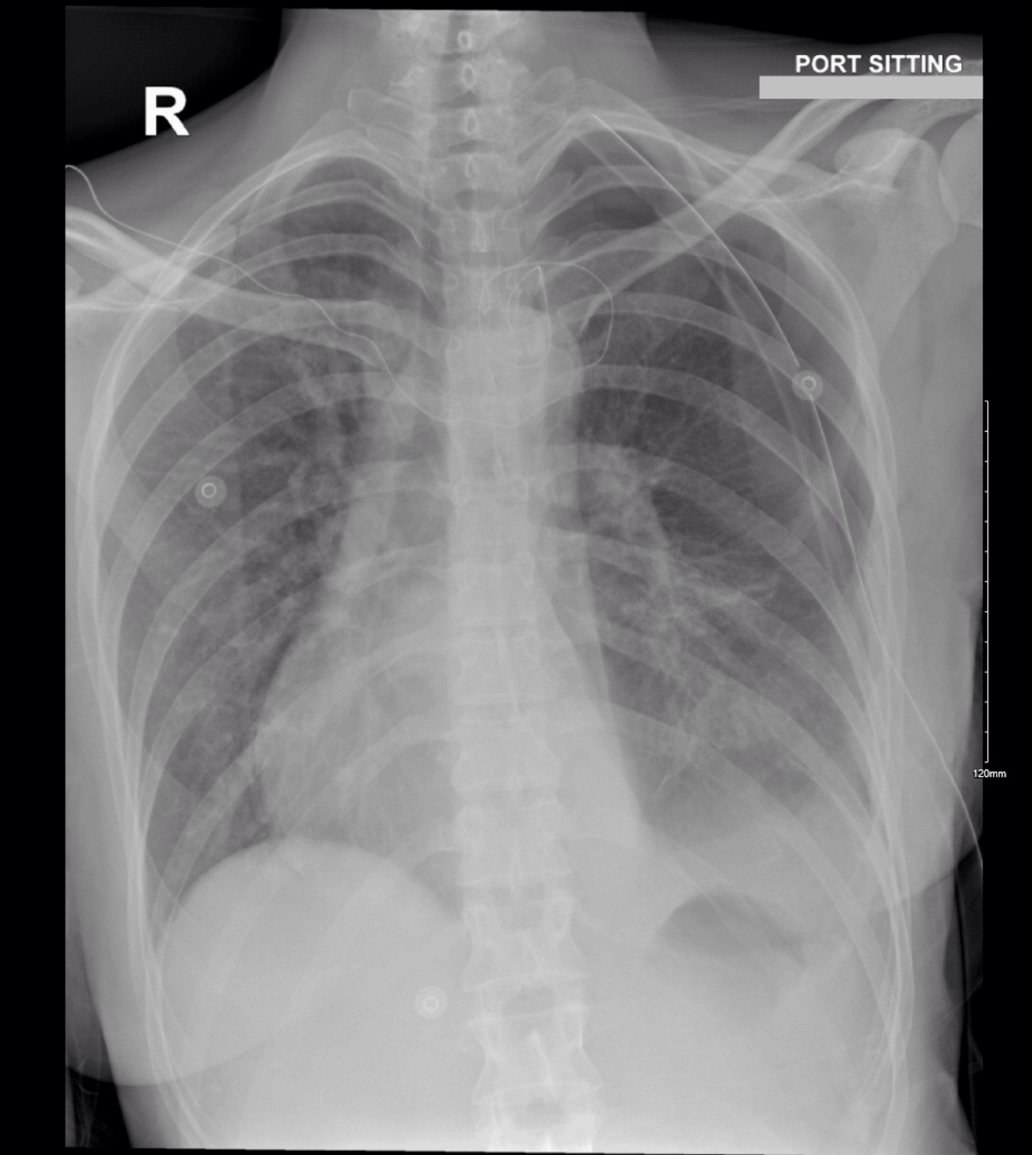
Postoperative chest X-ray demonstrating satisfactory expansion of the left lung

**Figure 8:**
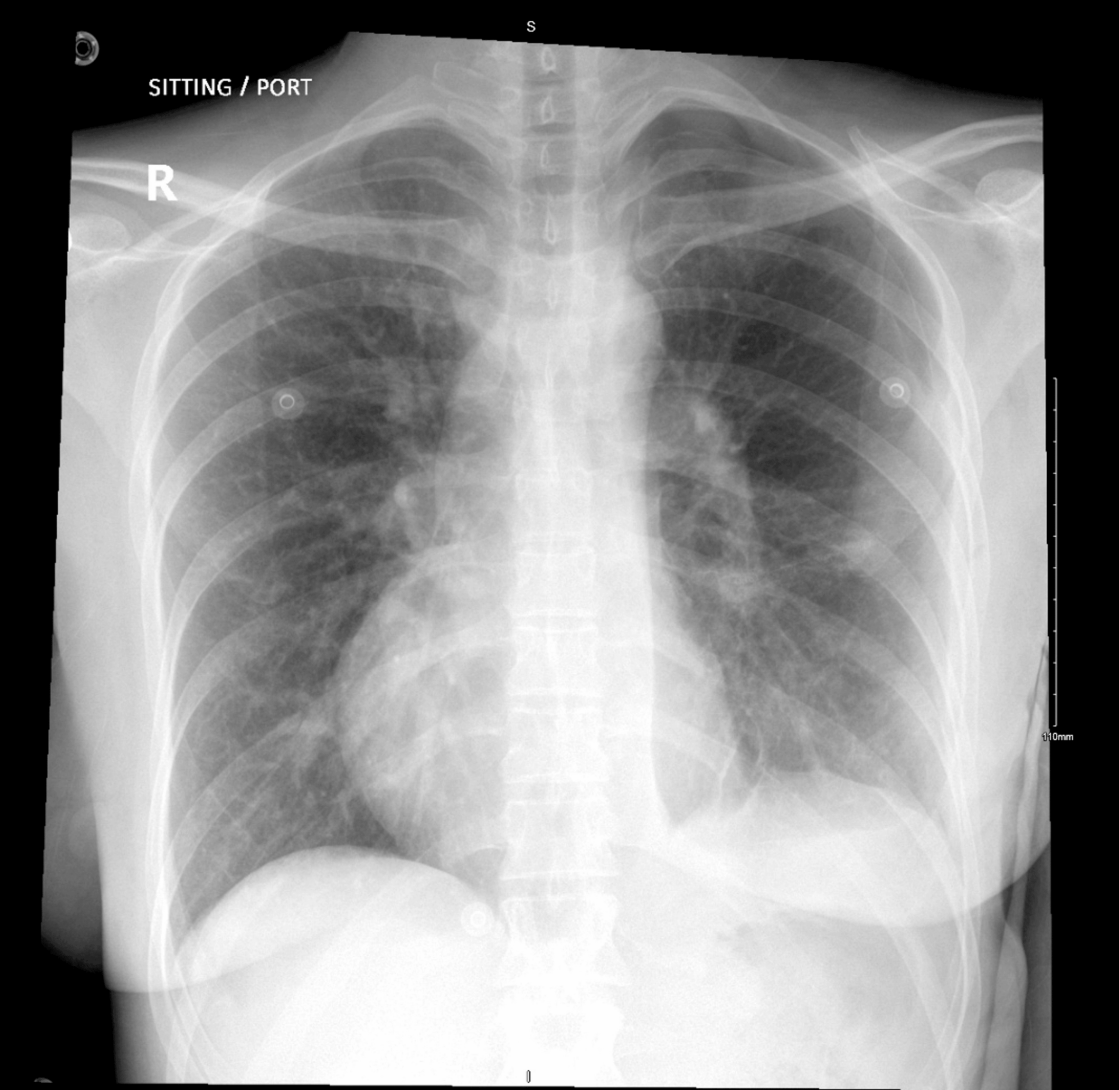
Chest X-ray on postoperative day one, after chest tube removal, showing a well-expanded lung with no complications

The patient was extubated at the end of the procedure and transferred to the recovery area in stable condition. A postoperative chest X-ray showed good left lung expansion with no evidence of air leak (Figure 7). The chest tube was removed on postoperative day one (Figure 8), and the patient remained stable and asymptomatic throughout her hospital stay. She was discharged home in good condition three days after surgery. 

At her one-week follow-up in the outpatient clinic, she was doing well. The pathology report confirmed benign lung bullae with areas of inflammation and fibrosis. She continued to follow up regularly with no complaints, and all subsequent imaging studies were unremarkable.

## Discussion

GB is a rare manifestation of irreversible lung parenchymal damage associated with COPD [1]. In 2010, approximately 11.7% of the global population was diagnosed with COPD, including 2.4% of individuals in Saudi Arabia [4,5]. GB commonly affects young male smokers and is typically characterized by a large bulla located in the upper lobes of the lungs. The protease-antiprotease theory suggests that exposure to cigarette smoke activates alveolar macrophages to release chemotactic factors, which in turn stimulate leukocytes to secrete neutrophil elastase. This cascade leads to unregulated elastase activity in the lungs, causing alveolar wall destruction. Under normal conditions, antiproteases, particularly alpha-1 antitrypsin, counteract these effects.

In individuals with an inherited deficiency of alpha-1 antitrypsin, even nonsmokers can develop elastolytic, panlobular emphysema [3]. GB results from persistent alveolar dilatation and progressive destruction of the lung parenchyma. It is defined as an air-filled cavity larger than 1 cm in diameter within emphysematous lung tissue. Fibrosis of the alveolar membranes and a reduction in alveolar surface area contribute to impaired gas exchange. These pathophysiologic changes may cause compression of the surrounding lung parenchyma and mediastinum, often mimicking pneumothorax on imaging [1,11].

Most cases of GBs are asymptomatic but may present with dyspnea or, rarely, hemoptysis. As space-occupying lesions, GBs disrupt normal respiration and gas exchange, leading to dyspnea, increased work of breathing, and exercise intolerance [3,7]. Roberts et al. described the radiologic criteria for GB 50 years after the first reported case. These criteria include the presence of GBs in one or both upper lobes occupying more than one-third of the hemithorax and causing compression of adjacent lung parenchyma [12]. Chest CT scanning is often required to differentiate between a pneumothorax and large bullae [3].

Unlike bullae, pneumothorax is defined as the presence of air in the pleural space and is classified based on whether it is spontaneous or traumatic. A spontaneous pneumothorax is further categorized as primary spontaneous pneumothorax in the absence of known lung disease or secondary in patients with underlying chronic lung conditions [13]. When the wall of a bulla is clearly visible, it may be misinterpreted as a pleural line on a chest X-ray, leading to misdiagnosis as a pneumothorax. The “double wall sign” on CT imaging helps differentiate between these conditions. It appears when air outlines both the inner and outer walls of a bulla, typically seen when the bulla wall is parallel to the chest wall. Absence of this sign supports the diagnosis of pneumothorax and can help avoid unnecessary chest tube placement. However, adjacent bullae may create a false-positive double wall sign. This can be avoided by carefully reviewing multiple CT images, ensuring there is no air in the pleural space and that the bulla wall is not parallel to the parietal pleura or chest wall [14].

GB can be managed surgically through stapled bullectomy, lung volume reduction using VATS, one-way endobronchial valves, or lung transplantation in advanced cases. Although there is no significant difference in mortality between treatment methods, surgical approaches are associated with lower risks of infection and pneumothorax. Bullectomy remains the treatment of choice for symptomatic GB, often leading to early improvement in dyspnea, hypoxemia, hypercapnia, pulmonary function, and overall quality of life. The most important determinant of postoperative improvement in lung function is the preoperative size of the bullae [8,9].

Potential complications of bullectomy include prolonged air leak lasting more than seven days, atrial fibrillation, postoperative mechanical ventilation, and pneumonia. Therefore, safer alternative approaches may be considered in selected cases [3]. Nevertheless, patients undergoing surgery often experience improved pulmonary function, better dyspnea scores, and enhanced quality of life [1]. Careful patient selection is essential, as resection of the bullae restores lung expansion, thereby improving respiratory mechanics and exercise tolerance [7].

Only a limited number of studies have reported cases of GBE (Table 1). Khan et al. and Samanta et al. described cases of iatrogenic pneumothorax following unnecessary chest tube insertion in patients with GB misdiagnosed as pneumothorax [13,15]. Another case involved the death of a 50-year-old male with bilateral GB, initially mistaken for pneumothorax. Bilateral chest tubes were inserted unnecessarily, resulting in a bronchopleural fistula and septic shock, despite subsequent bullectomy and antibiotic therapy [6]. In contrast, other reports - similar to our case - highlight patients who were accurately diagnosed with GBE using confirmatory CT before chest tube insertion. Some of these patients underwent surgical intervention with favorable outcomes and complete recovery [1,7,10,11,14-16].

**Table 1:** Summary of relevant studies on GBE mimicking pneumothorax COPD, chronic obstructive pulmonary disease; GBE, giant bullous emphysema

Author	Case no.	Age and sex	Clinical presentation	Chest tube insertion	Surgical intervention	Type of surgery	Outcome	Outcome details
Yousaf et al. (2020) [1]	1	64 M	Shortness of breath, nonproductive cough, wheezing, generalized weakness	No	No	-	Good	Improved modestly with oral prednisone
Ferreira Junior et al. (2019) [6]	2	50 M	Agitated, disoriented, intense respiratory distress	Yes	Yes	Right bullectomy	Death	Due to infectious complications
Napolitano et al. (2025) [7]	3	34 M	Progressive dyspnea, right-sided chest pain	No	Yes	Volume reduction surgery	Good	Procedure completed successfully without intraoperative complications
Louis et al. (2023) [10]	4	42 M	Recurring sharp right chest pain, shortness of breath	No	Yes	Right mini-thoracotomy	Good	Full recovery; resumed normal daily activities
Im et al. (2016) [11]	5	55 M	Shortness of breath, cough, increased sputum production	No	No	-	Good	Treated as COPD exacerbation; symptomatic improvement with steroids, antibiotics, and bronchodilators; discharged with home oxygen
Khan et al. (2021) [13]	6	51 M	Shortness of breath	Yes	No	-	Bad	Developed pulselessness after chest tube insertion; CPR and intubation required
Aramini et al. (2019) [14]	7	54 M	Intermittent shortness of breath, presyncope episodes	No	Yes	Left bullectomy	Good	Discharged in good condition on postoperative day 6
Samanta et al. (2022) [15]	8	48 F	Dyspnea, right-sided chest pain, generalized weakness	Yes	No	-	Good	Conservatively managed with inhaled tiotropium; discharged in good condition
Samanta et al. (2022) [15]	9	61 M	Productive cough, breathlessness	No	No	-	Good	Treated symptomatically for COPD exacerbation with bronchodilators, steroids, antibiotics, oxygen, and antihypertensives
Wang and Liu (2014) [16]	10	19 F	Acute pleuritic chest pain	No	Yes	Left bullectomy	Good	Bullectomy performed without complications; lung re-expanded successfully

## Conclusions

This study presents a case of a patient with GBE that was identified prior to chest tube insertion and managed surgically, resulting in a favorable outcome. The case highlights how GBs can clinically and radiologically mimic pneumothorax, potentially leading to inappropriate chest tube placement and serious complications. HRCT is the gold standard for distinguishing between the two conditions and is more accurate than chest X-ray. With proper evaluation, iatrogenic complications can be avoided, and appropriate management can be planned based on symptom severity, bulla size, and lung function. Further research is warranted to assess the long-term outcomes of surgical interventions and the accessibility of HRCT in emergency care settings.
